# CRISPR-based molecule-regulatory expression platform for specific immunotherapy of cancer

**DOI:** 10.3389/fonc.2024.1469319

**Published:** 2024-10-23

**Authors:** Tianying Zhan, Lu Tong, Linlin Wang, Jun Dong

**Affiliations:** Department of Clinical Laboratory Medicine, Guangdong Provincial Key Laboratory of Major Obstetric Diseases, Guangdong Provincial Clinical Research Center for Obstetrics and Gynecology, The Third Affiliated Hospital, Guangzhou Medical University, Guangzhou, China

**Keywords:** cancer immunotherapy, bladder cancer, miRNA, gene circuit, synthetic biology

## Abstract

**Introduction:**

Cancer is still a major challenge of human health. The abnormality of intracellular cancer-related signal pathways is an important mechanism for the occurrence of cancer.

**Methods:**

We used a molecular-senor to act on the endogenous signal molecules within the cell to redirect the abnormal signal flows in the cell to treat cancer. Based on CRISPR-dCas12f procedures, we combined aptamers and ribozymes to construct riboswitches, which served as molecular switches to reprogram sgRNAs, so that CRISPR-dCas12f redirected the intracellular anti-cancer signal flows after sensing specific input signal molecules. In addition, the activated molecular sensors and the inhibitory molecular sensors were constructed by combining transcription factors (VP64) and transcription inhibitors (KRAB) to specifically activate and inhibit target genes of interest.

**Results:**

Our experimental results showed that the molecular sensors that we designed and constructed specifically sensed the endogenous signal molecules and then redirect the cancer related signal networks of cancer cells. In addition, corresponding logic gates were constructed to distinguish cancer cells from normal cells and redirect anticancer signal flows to trigger specific cancer immunotherapy.

**Conclusion:**

The constructed molecular sensors constructed specifically recognized the signal molecules within the cell and redirected the endogenous signal pathway to reprogram the fate of cancer cells.

## Introduction

The core characteristics of miRNAs biogenesis and function have been identified and show great potential in the diagnosis and monitoring of various diseases, especially cancer (1–3). The miRNAs regulate gene expression, and their dysregulated and abnormal expression has been shown to have carcinogenic or tumor suppressor effects (4). Across the field of cancer biology, a large number of miRNAs have been identified and characterized, many of which are associated with cancer (5). These miRNAs further deepen our understanding of the genetic characteristics of cancer. The expressions of miRNAs vary in different tissues and cell types, which can effectively monitor the dynamic changes of disease development. In the progression of tumor, there are different miRNA expression profiles in different stages of tumor development. Although miRNAs provide essential information for tumor diagnosis and therapy, how to utilize these abundant endogenous miRNAs is still in infancy.

The bacterial type-II CRISPR coupled with the Cas9 provides a multifunctional tool for genome regulation (6). Wild-type or different effector proteins modified Cas9 would cut or epigenetically modify the target DNA sequence by the guidance of gRNA (7). Most CRISPR-Cas systems were found in prokaryotic genomes, but a growing number of reports suggested that sequences encoding CRISPR systems were also found in the genomes of giant phages (8). CRISPR-Cas12f, a member of the CRISPR-Cas family of systems derived from giant phages, is only 60-70 kDa and half the size of Cas9 or Cas12a, while it can edit target gene expression efficiently both in animal and plant cells (9). The gRNA structure of the Cas12f system is simple, which consists of only a hairpin structure and an antisense sequence, and is ideal for reprogramming with riboswitches. In summary, the Cas12f/dCas12f system facilitates a variety of biological and medical research as a revolutionary tool for manipulating the genome. To reach its full potential, the Cas12f/dCas12f system needs to be controlled according to specific cell types. Here, we created a CRISPR-Cas12f genome regulation platform through endogenous miRNA-mediated sgRNA release strategies. This platform can be controlled by specific endogenous or exogenous miRNAs or siRNAs (small interfering RNAs) and regulation the gene expression through a personalized positive/negative feedback loop.

## Methods

### Human primary T cells

We purchased the human primary T cells from OriBiotech (Shanghai, China) and cultured them in RMPI1640 supplemented with 30 units/mL IL-2 (Beijing T&L Biotechnology CO, LTD) and 5% FBS. The T cells were amplified with Dynabeads^®^ Human T-Activator CD28/CD3 (Gibco^®^, 11163D). Dynabeads^®^ were added at a cell to bead ratio of 1:1. The beads were separated with a magnet after activation for 3 days, and the cells were cultured for at least a week before they were used for other experiments.

### Cell lines and cell culture

HEK293t cells and Bladder cancer cells T24, UMUC3 were cultured in DMEM (Gibco^®^, Life Technologies, Carlsbad, CA) supplemented with 5% fetal bovine serum (FBS), 100U/ml penicillin/streptomycin in 37°C with 5% CO2.

### *In vitro* cytotoxicity assay

The transfected cancer cells were mixed with human T cells (effector cells) for 24 h in the presence of CAN-TE (0-200 nM) at 37°C. The assay was carried out 96 well flat-bottom plates. After 24 h incubation, LDH release assay with CytoTox 96_ Non-Radioactive Cytotoxicity Assay Kit (Promega, Madison, WI; catalog #G1780) were used to measure the cytotoxicity *in vitro (* 10).

### Statistical analysis

Data were summarized as the mean ± SD. Significance tests were performed using SPSS statistical software, version 20.0 (SPSS, Chicago, IL, USA). Statistical significance was determined using Student’s *t*-tests or analysis of variance. Value of P < 0.05 was considered to be statistically significant.

## Results

### Re-engineer of gRNA for sensing miRNA

**Figure 1:**
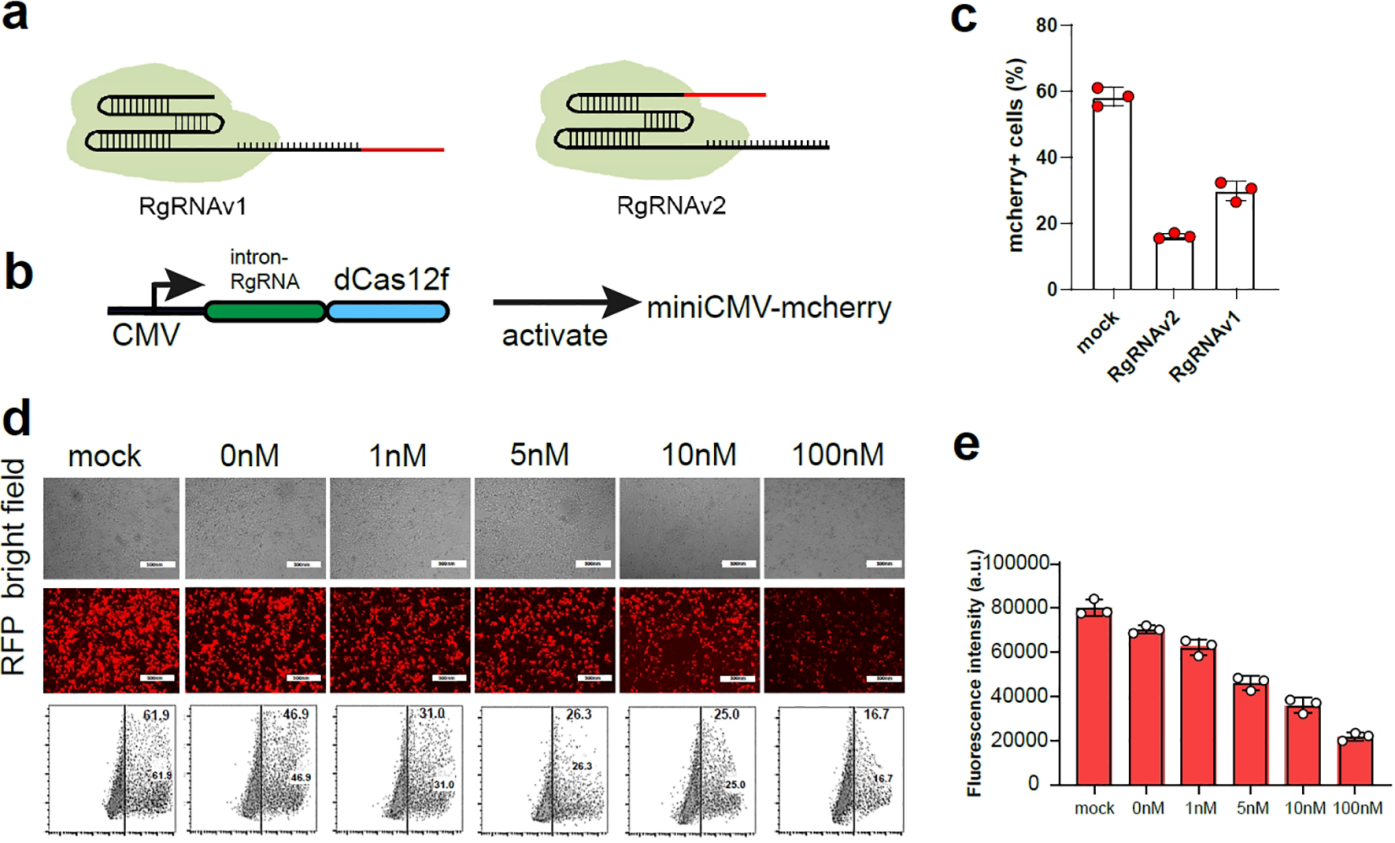
The construction the RgRNA for sensing miRNAs. **(A)** In the design of RgRNAv1, the miRNA binding site was connected to the 5’ end of the sgRNA (left), and the miRNA binding site was connected to the 3’ end of the sgRNA in the design of RgRNAv2 (right). **(B)** The schematic diagram of expression pattern for RgRNA mediated CRISPR-dCas12f/VPR. **(C)** The ratio of mcherry+ cell in 293t cells expressing miniCMV-mcherry transfected with the plasmid of control gRNA, RgRNAv1 and RgRNAv2 mediated CRISPR-dCas12f/VPR, respectively. **(D)** The representative fluorescence figure of 293t cells co-transfected with RgRNAv2 mediated CRISPR-dCas12f/VPR, miniCMV-mcherry and different dose of miRNA-1 mimics. **(E)** The ratio of mcherry^+^ cells of 293t cells co-transfected with RgRNAv2 mediated CRISPR-dCas12f/VPR, miniCMV-mcherry and different dose of miRNA-1 mimics.

A critical component of the miRNA-regulatory gRNA switch strategy was to construct the RgRNA, in which the sgRNA is reprogrammed by miR-BS. The miR-BS can match the target miRNA/siRNA and degrade the RgRNA through RNA-induced silencing complex (11–13). In our design, two versions of RgRNA have been constructed via editing miR-BS into 3’ of gRNA (RgRNAv1), and encoding miR-BS into 5’ of gRNA (RgRNAv2), respectively (**Figure 1A**). We inserted the designed RgRNAs as the introns into the Cas12f/dCas12f-VP64 expression cassettes, and used CMV promoters to drive their expression which was used to activate the mcherry expression driven by minimal promoter (**Figure 1B**). We used the binding site of miRNA-1 to re-engineer the gRNA of Cas12f to verify whether such gRNA re-engineered strategy is feasible. Theoretically, when the target miRNA binds to the binding site on RgRNA, the miRNA will degrade the gRNA and the Cas12f/dCas12f-VP64 system will fail to work properly. We found that both RgRNAv1 and RgRNAv2 could be degraded by the miRNA-1 mimics, but miRNA-1 showed stronger inhibitory effect on RgRNAv1 mediated Cas12f/dCas12f-VP64 (**Figure 1C**). Therefore, RgRNAv2 was used for further research.

Next, we continued to investigate whether RgRNAv2-mediated CRISPR-dCas12f could be regulated by different dose of target miRNA-1 mimics. We used RgRNAv1 to mediate CRISPR-dCas12f/VPR to stimulate miniCMV-mcherry expression in 293t cell. We found that the expression of mcherry decreased with the increase of miRNA-1 transfection, indicating that the more miRNA will result in the more obvious the degradation of RgRNA (**Figures 1D, E**).

### Design of the miRNA-regulatory CRISPR-Cas12f platform

**Figure 2:**
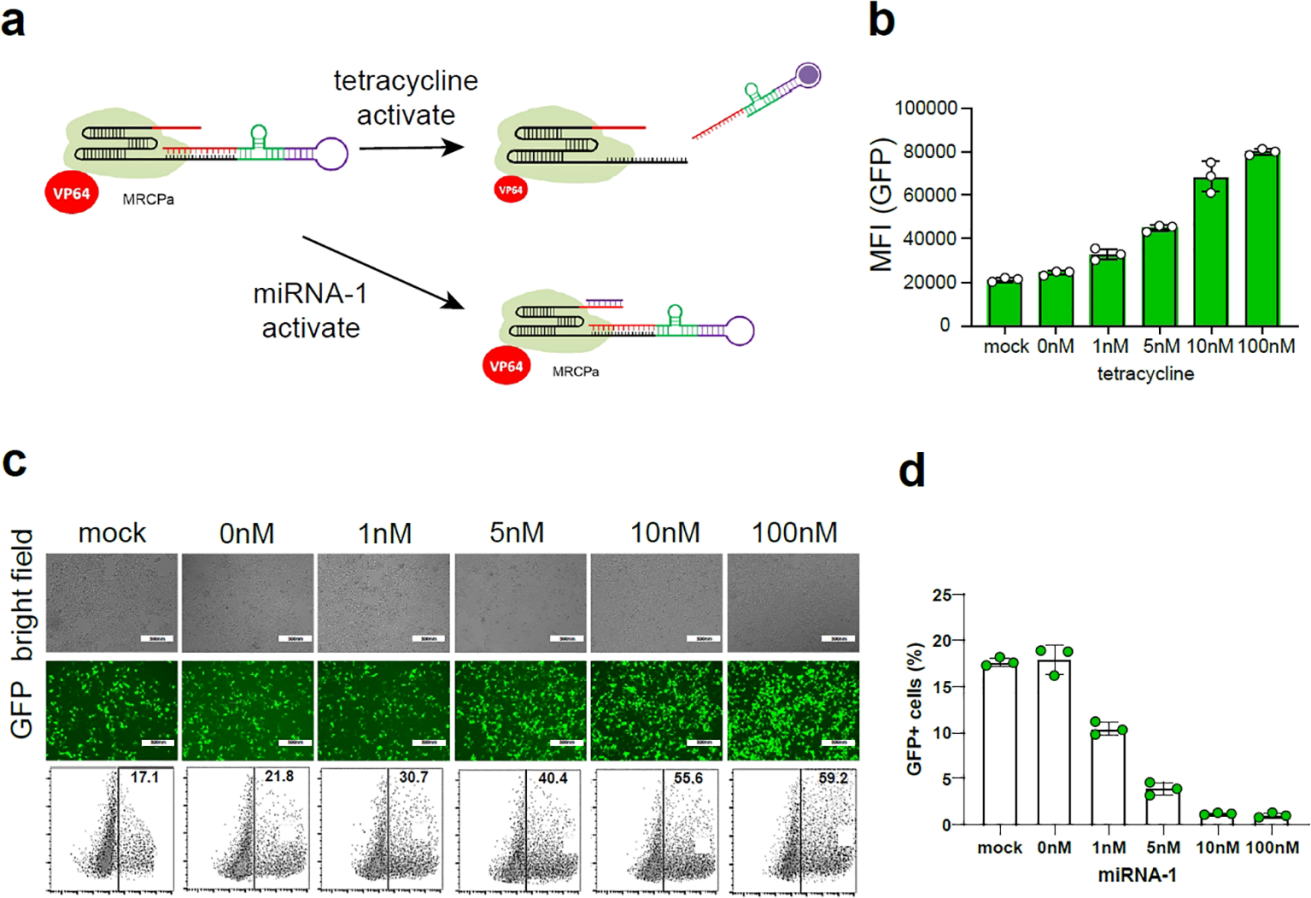
The construction of MRCP. **(A)** The schematic diagram of MRCP: The aptamer was combined with the ribozyme to construct a riboswitch, and the riboswitch was connected to the 5’ end of the sgRNA; The miRNA binding site was connected to the 3’ end of the sgRNA. **(B)** The fluorescence intensity of GFP^+^ cells of 293t cells co-transfected with MRCP, miniCMV-GFP and different dose of tetracycline. **(C)** The representative fluorescence figure of 293t cells co-transfected with MRCP, miniCMV-GFP and different dose of tetracycline. **(D)** The ratio of GFP^+^ cells of 293t cells co-transfected with MRCP, miniCMV-GFP and different dose of miRNA-1 mimics.

We sought to design a molecule-regulatory CRISPR-dCas12f/VPR-based platform (MRCP) via the co-regulation of miRNA and signaling molecules. First, we used aptamer and ribozymes to re-engineer the gRNA of CRISPR-Cas12f for sensing target small molecule signals (14). We inserted the sequence of aptamer and ribozyme to the 5’ of the gRNA (**Figure 2A**). In the proof-of-principle assay, we used tetracycline aptamer to re-engineer gRNA to mediate CRISPR-dCas12f/VPR for activating the GFP expression driven by miniCMV. We found tetracycline can switch on the gRNA re-engineered by tetracycline aptamers and promote the expression of GFP. In addition, the expression of GFP increased correspondingly with increased dosage of tetracycline (**Figures 2B, C**). Remarkably, in the absence of tetracycline, there was still some expression of GFP, indicating that such re-engineering strategy using aptamer and ribozyme cannot silence gRNA function completely.

To further eliminate background activation of the re-engineered gRNA using aptamer and ribozyme, we used the miRNA binding site to insert the 3’ of gRNA (**Figure 2A**). In the proof-of-principle assay, we used miRNA-1 binding site to re-engineer gRNA to mediate CRISPR-dCas12f/VPR for activating the GFP expression driven by miniCMV. In the absence of tetracycline, the expression of GFP decreased correspondingly with increased dosage of miRNA-1 mimics.

### Construction and characterization of tumor-specific MRCP

We proposed a tumor-specific MRCP for regulating the expression of the target genes, which holds significant promise in the realm of precision gene therapy for tumor. miR-34a has been reported to be associated with the expression of wild-type p53 (15). In normal cells, miR-34a exhibit high expression levels, while cells undergo deterioration and progress towards a tumor cell phenotype, the expression of miR-34a is often lost (16). β-catenin is an important tumor-associated transcription factor, which is highly expressed in tumor cells and low expressed in normal cells (17). Here, we sought to re-engineer the 5’ of gRNA with β-catenin aptamer, which can switch on MRCP in interaction with β-catenin for activating the expression of target genes. In addition, we sought to re-engineer the 3’ of gRNA with miR-34a binding site, which can switch off MRCP under the action of miR-34a for silencing the function of CRISPR-dCas12f/VPR.

**Figure 3:**
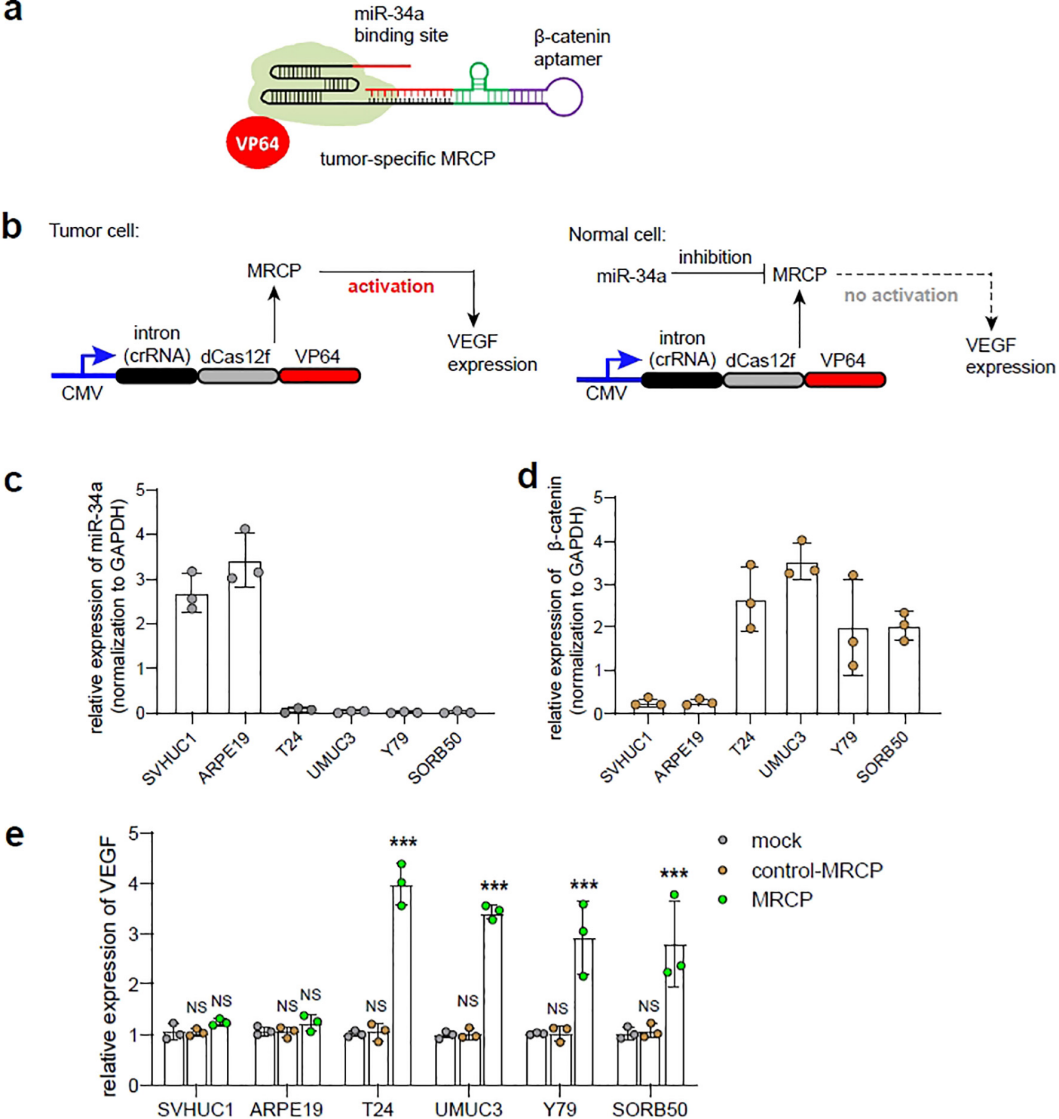
The construction of tumor-specific MRCP. **(A)** The schematic diagram of the tumor-specific MRCP. The β-catenin aptamer was combined with the ribozyme to construct a riboswitch, and the riboswitch was connected to the 5’ end of the sgRNA, and the miR-34a binding site was connected to the 3’ end of the sgRNA. **(B)** The schematic diagram of the tumor-specific MRCP working in tumor cells (left) and normal cells (right). **(C)** The relative expression of miR-34a in SVHUC1, ARPE19, T24, UMUC3, Y79 and SORB50. **(D)** The relative expression of β-catenin in SVHUC1, ARPE19, T24, UMUC3, Y79 and SORB50. **(E)** The relative expression of VEGF in SVHUC1, ARPE19, T24, UMUC3, Y79 and SORB50. Mock group: the wild type cells. control MRCP group: The cells transfected with the MRCP that cannot activate any genes. MRCP group: The cells transfected with the MRCP that designed for activating VEGF expression. (***<0.001).

We hypothesized that in tumor cells, highly expressed β-catenin will switch on MRCP and release the CRISPR-dCas12f/VPR system that regulates genes of interest expression within cells (**Figure 3A**). In normal cells, although there is low expression of β-catenin that partially releases the working CRISPR-dCas12f/VPR system, high expression of miR-34a predominates the degradation of gRNA, which silencing MRCP to protect normal cells from being affected (**Figure 3B**).

In the proof-of-principle assay, we designed the tumor-specific MRCP to regulate the endogenous VEGF expression, and test in cell models *in vitro*. The expression levels of miR-34a and β-catenin in cells were measured by qPCR assay, and found that the miR-34a exhibited high expression in normal cells (SVHUC1, ARPE-19), while β-catenin was significantly high expressing in tumor cells (T24, UMUC3, Y79 and SO-RB50) as expected (**Figure 3C**). Next, we used qPCR to measure the expression level of VEGF by MRCP and determined whether tumor-specific MRCP can regulate gene expression of tumor cells specifically. Interesting, compared with normal cells transfected with MRCP for VEGF and the cells transfected with non-functional MRCP, the tumor cells transfected with MRCP for VEGF exhibited relatively high expression of VEGF (**Figure 3D**). Remarkably, the normal cells transfected with MRCP for VEGF did not show significant VEGF activation.

Overall, the tumor-specific MRCP described here does have the potential to specifically regulate the expression of specific genes within tumor cells.

### Tumor-specific MRCP was used to inhibit the malignant phenotype of tumors

To investigate the efficiency of tumor-specific MRCP regulating endogenous gene expression in tumor cells for inhibiting the malignant phenotype, we constructed and evaluated MRCP to regulate cyclin-dependent kinase inhibitor 1A (CDKN1A) and Bax expression in tumor cells. As reported, up-regulating the CDKN1A expression strongly inhibits cell proliferation, and Bax is a key gene that can induce apoptotic cell death (18, 19). We selected the *in vitro* cell model of bladder cancer to determine the inhibition of MRCP on the malignant phenotype of the tumor.

**Figure 4:**
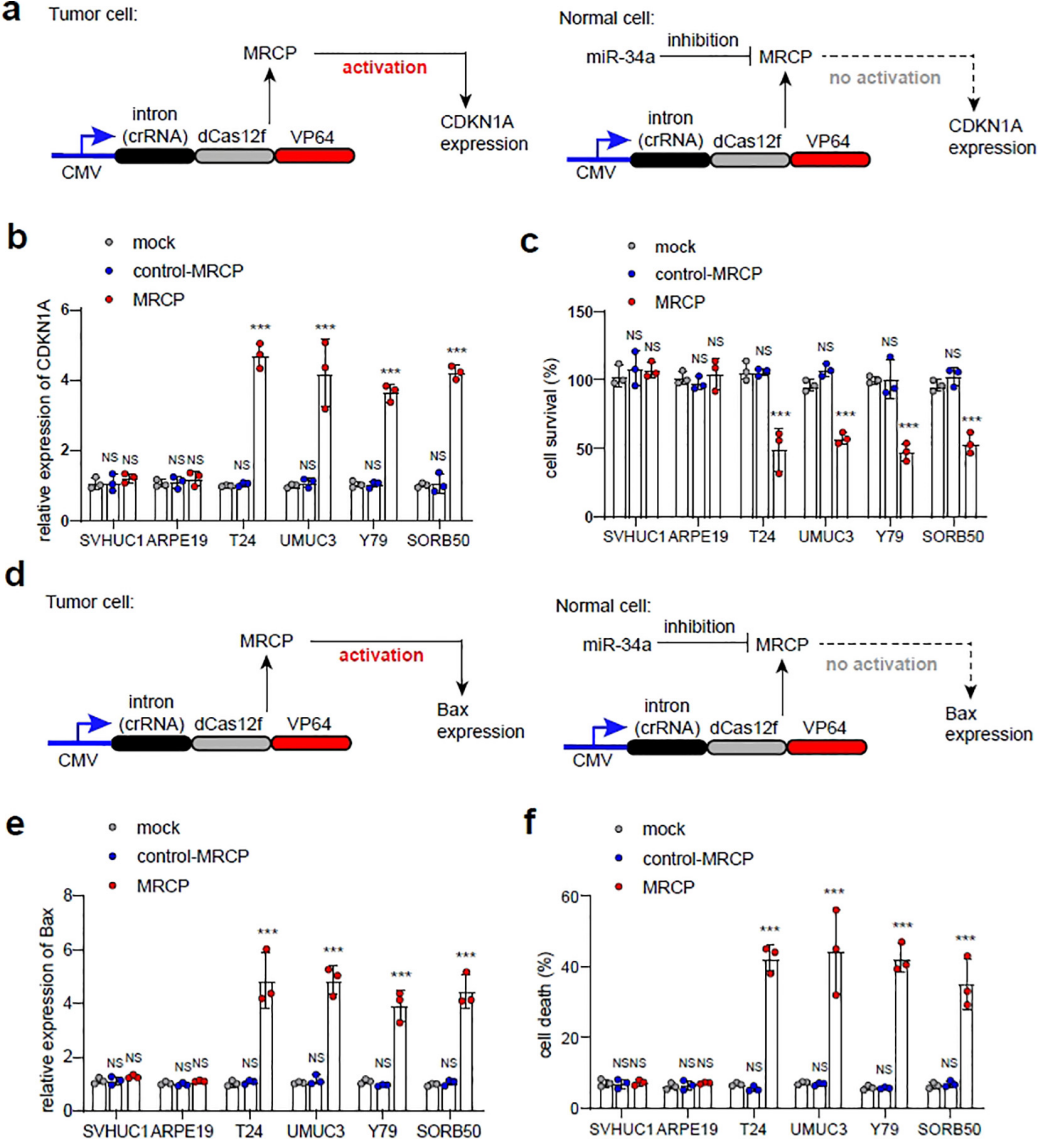
Inhibiting malignant phenotype of tumor cells using tumor-specific MRCP. **(A)** Up-regulation of CDKN1A using tumor-specific MRCP in tumor cells specifically. **(B, C)**: Mock group: the wild type cells. control MRCP group: The cells transfected with the MRCP that cannot activate any genes. MRCP group: The cells transfected with the MRCP that designed for activating CDKN1A expression. The relative expression level of CDKN1A in SVHUC1, ARPE19, T24, UMUC3, Y79 and SORB50 **(B)**. The cell survival of SVHUC1, ARPE19, T24, UMUC3, Y79 and SORB50 in different groups **(C)**. **(D)** Up-regulation of Bax using tumor-specific MRCP in tumor cells specifically. **(E, F)** Mock group: the wild type cells. control MRCP group: The cells transfected with the MRCP that cannot activate any genes. MRCP group: The cells transfected with the MRCP that designed for activating CDKN1A expression. The relative expression level of Bax in SVHUC1, ARPE19, T24, UMUC3, Y79 and SORB50 **(E)**. The ratio of cell death in SVHUC1, ARPE19, T24, UMUC3, Y79 and SORB50 in different groups **(F)**. (***<0.001).

First, we constructed the tumor-specific MRCP for up-regulation of CDKN1A specifically (**Figure 4A**). As expected, the MRCP can activate the CDKN1A expression in tumor cells significantly, while not in normal cells (**Figure 4B**). Accordingly, we found that the cell growth of tumor cells transfected with MRCP for CDKN1A was significantly inhibited, while the growth of normal cells was not significantly affected (**Figure 4C**). Next, we sought to determine whether MRCP can promote tumor apoptosis by regulating bax gene expression (**Figure 4D**). As expected, the customized MRCP for bax gene can specifically overexpress Bax gene in tumor cells and induce apoptosis of tumor cells (**Figures 4E, F**).

### MRCP mediated genes expression for triggering immune response of killing bladder cancer cell *in vitro*

**Figure 5:**
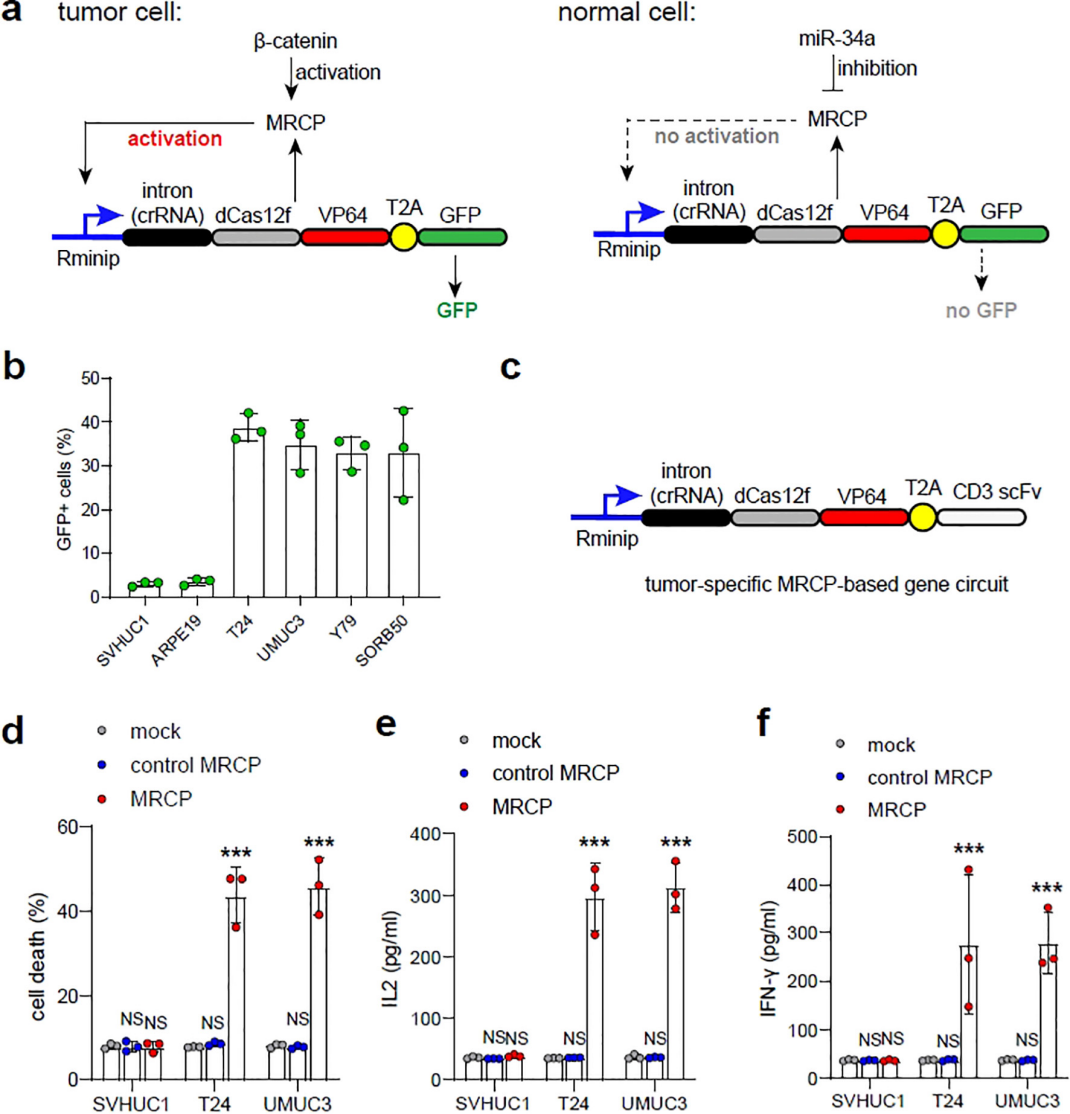
Triggering immunotherapy using tumor-specific MRCP-based gene circuit for treating bladder cacner. **(A)** The schematic diagram of the tumor-specific MRCP-based gene circuit expressing GFP. **(B)** The ratio of GFP cells in SVHUC1, ARPE19, T24, UMUC3, Y79 and SORB50 transfected with the plasmid of the tumor-specific MRCP-based gene circuit expressing GFP. **(C)** The schematic diagram of the tumor-specific MRCP-based gene circuit expressing CD3 scFv. **(C, D)** Mock group: the wild type cells. control MRCP group: The cells transfected with the MRCP that cannot activate any genes. MRCP group: The cells transfected with the MRCP that designed for activating CDKN1A expression. The ratio of cell death in SVHUC1, ARPE19, T24, UMUC3, Y79 and SORB50 co-cultured with PBMCs **(D)**. The expression of IL-2 in SVHUC1, ARPE19, T24, UMUC3, Y79 and SORB50 co-cultured with PBMCs **(E)**. The expression of INF-γ in SVHUC1, ARPE19, T24, UMUC3, Y79 and SORB50 co-cultured with PBMCs **(F)**. (***<0.001).

To further test the feasibility of tumor-specific MRCP, we first designed a MRCP-based gene expression pattern that contained two components: an expression vector consisted of the redesigned minimal-promoter (Rminip) driving the intron-RgRNA and dCas12f-VP64 encoding cassette expression, and an expression vector contained the expression cassette of GFP (MRCP-GFP) (**Figure 5A**). This min-promoter contains only one TATA-box and multiple binding sites of CRISPR-dCas12f-VP64 in tandam (20)(**Figure 5A**). We validated the MRCP-GFP in *in vitro* cell models. We found that compared with normal cells, the tumor cells transfected with MRCP-GFP exhibited robust GFP expression (**Figure 5B**). The tumor-specific MRCP-based gene expression pattern was used to drive the expression of the membrane-anchored anti-CD3 single-chain variable fragment (CD3-scFv), which induced T cells to kill cancer cells specifically (10) (**Figure 5C**). We found that the tumor-specific MRCP-based gene circuit worked in bladder cancer cells efficiently. The expression of CD3-scFv driven by gene circuit could induce T cells to kill tumor cells effectively (**Figure 5D**). Next, the expression levels of INF-γ and IL-2 in the supernatant of T cells co-cultured with T24 cells were measured by ELISA to assess the level of T cell activation. We found that the tumor-specific MRCP-based gene circuit could only activate T cells in bladder cancer cell, but not in normal bladder epithelial cells (**Figures 5E, F**).

## Discussion

Understanding the overall changes in cancer at the molecular level is crucial for the development of precise tumor therapies. Cancer is the result of the complex interplay of multiple genes, making it challenging to achieve the specific identification of tumor cells with a single target. In comparison to normal cells, many molecules such as transcription factors and RNA in tumor cells exhibit aberrant expression or loss of expression. Therefore, precise cancer therapy requires the simultaneous recognition of various signals. By comprehensively analyzing multiple cancer signaling molecules, the design of artificial gene circuits that can specifically identify and distinguish particular cell types by concurrently processing multiple input signals shows promising prospects in various human diseases, including tumors. The construction of artificial gene circuits for multi-signal recognition has been reported to effectively identify specific types of tumors. In contrast, traditional gene therapies, which rely on a single-gene treatment strategy, often fail to achieve the desired anti-cancer effects.

Here, we have successfully designed a compact CRISPR platform that can be regulated by specific miRNA or siRNA. In our design, the miRNA-BS is incorporated into the gRNA of CRISPR-Cas12f, which is complementary to a specific miRNA, resulting in the generation of RgRNA. RgRNA can be bound by homologous miRNA and trigger its cleavage, preventing the action of CRISPR-Cas12f/dCas12f. In the absence of homologous miRNA, RgRNA exhibits gene-editing effects on par with wild-type gRNA, guiding Cas12f. We have developed the MRCP system, enabling the detection and regulation of cell behavior under the control of endogenous or synthetic miRNA. Furthermore, the establishment of a positive feedback gene expression pattern based on MRC can be utilized to sense the cell type-specific activity of miRNA, distinguishing normal cells from bladder cancer tumor cells for targeted cancer therapy. In summary, MRCP represents a novel biotechnological tool dependent on miRNA, allowing for the specific monitoring and regulation of cell types. It holds great potential for the development of targeted therapies in the direction of specific human diseases.

Many miRNAs are expressed exclusively in specific cell types or different stages of cell development. The MRCP system we report can leverage this miRNA information through the CRISPR system to accurately regulate cellular behavior in specific cell types. This includes activities such as genome editing, precise base editing, epigenetic modifications, and transcriptional control. Furthermore, the positive feedback gene expression pattern based on MRCP can be used to drive the expression of different transgenes, such as toxins for killing specific cells or Cre recombinase for lineage tracing. Regulating the expression of the CRISPR system using tissue-specific promoters can also achieve expression in specific cell types. However, this strategy is challenging to implement effective cell control in the complex *in vivo* environment. In contrast, MRCP, which operates specifically based on miRNA regulation, can adapt well to various complex intracellular environments. Therefore, we anticipate that MRCP will have superiority in addressing complex intracellular environments compared to strategies relying on tissue-specific promoters in the context of *in vivo* complexities.

## Data Availability

The raw data supporting the conclusions of this article will be made available by the authors, without undue reservation.
